# Synthesis, Physicochemical Characterization, and Cytotoxicity Assessment of Rh Nanoparticles with Different Morphologies-as Potential XFCT Nanoprobes

**DOI:** 10.3390/nano10112129

**Published:** 2020-10-27

**Authors:** Yuyang Li, Giovanni M. Saladino, Kian Shaker, Martin Svenda, Carmen Vogt, Bertha Brodin, Hans M. Hertz, Muhammet S. Toprak

**Affiliations:** Biomedical and X-ray Physics, Department of Applied Physics, KTH Royal Institute of Technology, SE 10691 Stockholm, Sweden; yuyangli@kth.se (Y.L.); saladino@kth.se (G.M.S.); kiansd@kth.se (K.S.); martin.svenda@biox.kth.se (M.S.); carmenma@kth.se (C.V.); berthab@kth.se (B.B.); hans.hertz@biox.kth.se (H.M.H.)

**Keywords:** polyol synthesis, rhodium nanoparticles, surfactants, role of additives, morphology control, toxicity, bio-imaging, X-ray fluorescence, contrast agent, XFCT

## Abstract

Morphologically controllable synthesis of Rh nanoparticles (NPs) was achieved by the use of additives during polyol synthesis. The effect of salts and surfactant additives including PVP, sodium acetate, sodium citrate, CTAB, CTAC, and potassium bromide on Rh NPs morphology was investigated. When PVP was used as the only additive, trigonal NPs were obtained. Additives containing Br^−^ ions (CTAB and KBr) resulted in NPs with a cubic morphology, while those with carboxyl groups (sodium citrate and acetate) formed spheroid NPs. The use of Cl^−^ ions (CTAC) resulted in a mixture of polygon morphologies. Cytotoxicity of these NPs was evaluated on macrophages and ovarian cancer cell lines. Membrane integrity and cellular activity are both influenced to a similar extent, for both the cell lines, with respect to the morphology of Rh NPs. The cells exposed to trigonal Rh NPs showed the highest viability, among the NP series. Particles with a mixed polygon morphology had the highest cytotoxic impact, followed by cubic and spherical NPs. The Rh NPs were further demonstrated as contrast agents for X-ray fluorescence computed tomography (XFCT) in a small-animal imaging setting. This work provides a detailed route for the synthesis, morphology control, and characterization of Rh NPs as viable contrast agents for XFCT bio-imaging.

## 1. Introduction

Like noble metals Pt and Pd, Rh nanomaterials have caught increasing attention due to applications in catalysis, photonics, and biosensors [1,2,3,4]. Physical and chemical properties of nanomaterials are strongly related to the size and morphology of particles. For instance, catalytic properties are associated with the active sites, which is reflected by the spatial shape and exposed crystal facets of metallic nanoparticles (NPs) [5]. In general, a high specific surface area contributes to a high catalytic activity, due to high-index crystal planes showing high binding affinity [5,6]. Likewise, nanomaterials also reveal an inherent correlation between toxicity and their surface properties within biological applications [7,8,9,10]. The specific surface-exposed groups could be the reactive sites inducing superoxide radical formation, as major reactive oxygen species (ROS) which are cytotoxic [10]. The hydrophilicity–hydrophobicity, or lipophilicity–lipophobicity, of nanomaterials functionalized by various groups also plays a key role in the toxicity and biocompatibility. Metallic nanomaterials, including Ag and Au, show biocompatibility with a strong dependence on particle size, morphology, and surface properties [11]. The toxicity of Rh NPs as a function of their particle morphology has been rarely discussed in the literature [12]. It is, therefore, scientifically important to explore Rh NPs’ morphological effects in connection with their potential biological applications.

Enormous efforts have been devoted to tune the synthesis methods in order to control the morphology and surface properties of metallic NPs. Surface capping agents such as salts and surfactants have shown significant effects on directing the growth of nanocrystals. A diverse set of morphologies of Ag and Au NPs have been reported using different capping agents. For example, PVP preferentially adsorbs on and reduces the growth rate of the Ag(100) surface, while PVP passivates the Au(111) surface. Halides anions like Br^−^ also adsorb on the Pd(100) surface. Furthermore, citrate is able to passivate the Pd(111) surface [8]. Yu et al. [13] reported on the surface energy of noble metals Rh, Pt, Pd, and Au that is different for various nanocrystal shapes, where Rh showed the highest surface energy for the crystal planes with the same index. The difference in surface energy values is significant (about five–six times higher) when compared to Au, which makes Rh unique as a catalyst. However, if necessary, it is possible to passivate the catalytic activity with selective surface-adsorbed ligands. Furthermore, this extraordinarily high surface energy makes it rather challenging to have a good shape control for Rh [13]. There are very few studies, though, focusing on the morphology control of Rh particles. Br^−^ ions, for instance, were reported to passivate the (100) plane in Rh nanocrystals [14]. The morphology control of metallic Rh NPs during typical polyol synthesis was demonstrated by replacing the Rh salt precursors with organometallic Rh Rh_2_(COOCF_3_)_4_, and the solvent with other polyols such as diethylene glycol, or tri-ethylene glycol [1,15]. 

We recently demonstrated the potential use of Rh NPs as X-ray fluorescence computed tomography (XFCT) contrast agents [16,17]. CCK-8-based cytotoxicity assays of triangular Rh NPs based on two cells lines, murine macrophages and human-derived ovarian cancer cells, showed toxicity for doses above 40 mg/L [17]. Further research is needed to understand the toxicity induced by Rh NPs. In this work, we present a systematic study on the effect of halide ions and carboxyl salts on the morphology of Rh NPs by one-pot polyol synthesis. The morphological and surface properties and cytotoxicity of morphologically different Rh NPs are studied and discussed, finally demonstrating the performance of Rh NPs in a small-animal XFCT imaging scenario.

## 2. Materials and Methods 

### 2.1. Materials

Rhodium (III) chloride hydrate (RhCl_3_·xH_2_O, Rh 38.5–45.5%), Ethylene glycol (EG, HOCH_2_CH_2_OH, >99%), Poly(vinyl pyrrolidone) (PVP, C_2_H_2_N(C_6_H_9_NO)_n_C_13_H_10_NS_2_, average MW = 55 kDa), Potassium bromide (KBr, >99%), Sodium Acetate trihydrate (NaAc; CH_3_COONa·3H_2_O, >99%), Hexadecyltrimethylammonium bromide (CTAB, CH_3_(CH_2_)_15_N(Br)(CH_3_)_3_, >99%), Hexadecyltrimethylammonium chloride (CTAC, CH_3_(CH_2_)_15_N(Cl)(CH_3_)_3_, >99%), and Sodium citrate dihydrate (NaCit; HOC(COONa)(CH_2_COONa)_2_·2H_2_O, 99%) were obtained from Sigma Aldrich, Germany. Hydrochloric acid, Sodium hydroxide, and solvents, including acetone and ethanol, were of analytical grade and were obtained from Sigma Aldrich, Sweden. All chemicals were used without further purification.

### 2.2. Synthesis of Rh NPs 

The synthesis was based on a modified polyol reduction process [17]. In a typical synthesis, 20 mL EG is used as solvent. The halide salts, surfactants (molar ratio of Rh precursor/additive 1:1), and PVP (4 mmol in repeating units) were dissolved in EG in a glycerol bath. A series of reactions were performed for tuning the morphology of Rh NPs, by using the following additives in combination with PVP: Na-Ac, Na-Cit, CTAB, KBr, and CTAC. Details of all the synthesized samples are given in Table 1. After the addition of 0.2 mmol RhCl_3_, the solution was heated to 85 °C, where nucleation was initiated, monitored from the darkening of the solution’s color. After 15 min, the temperature was ramped up to 115 °C, where it was kept for 2 h, followed by quenching. The NPs obtained were centrifuged and washed three times through precipitation with acetone and re-dispersion in deionized water (DIW). Thereafter, they were dispersed in DIW for further characterizations.

### 2.3. Characterization Methods 

The morphology and crystallinity of Rh samples were characterized by transmission electron microscopy (TEM) (JEM-2100F, 200 kV, JEOL Ltd., Tokyo, Japan). The TEM samples were prepared by transferring a ∼10 µL droplet of colloidal suspension onto a carbon-coated copper grid (Ted-Pella, CA, USA), and drying for 12 h. Particle size was measured by ImageJ by counting around 200 NPs in different fields of view on several TEM micrographs. Dynamic light scattering (DLS, Malvern Nano-ZS90, Malvern, UK) was used to measure the hydrodynamic size distribution of the colloidal suspension of as-prepared Rh NPs dispersed in DI water, at pH 7. Surface charge (Zeta potential) was also measured on the same samples using the same system under AC field. Inductively coupled plasma-optical emission spectroscopy (ICP-OES) (iCAP 6000 series, Thermo Scientific, Waltham, MA, USA) was used for the determination of the elemental Rh concentration in the colloidal suspensions prior to cytotoxicity tests. Ultraviolet–visible spectroscopy (UV–Vis) (NanoPhotometer NP80, Implen, Munich, Germany) was used to measure the absorption spectroscopy of Rh samples synthesized with different additives. Fourier-transform infrared spectroscopy (FT-IR, Thermo Scientific Nicolet iS20, Stockholm, Sweden) was used to obtain FT-IR spectra in transmission mode, on KBr pellets (2 mg sample in 100 mg KBr), in the spectral range of 4000–400 cm^−1^. Thermal gravimetric analysis (TGA) was performed on dried samples in the temperature range of 30–700 °C, using the TGA 550 system (TA instruments, Sollentuna, Sweden). 

### 2.4. In Vitro Toxicity 

Before proceeding with the in vitro toxicity analysis, Rh NP suspensions were tested for lipopolysaccharides (LPS) contamination [18] with PTS cartridges with a sensitivity of 0.005 EU/mL (Endosafe-PTS™, Charles River) following the manufacturer’s instructions. All the NPs suspensions had LPS values below the maximum admissible limit of 0.1 EU/mL [19]. 

Toxicity tests were performed on two different cell lines which were RAW 264.7 (murine macrophages, 91062702-1VL, SigmaAldrich, Stockholm, Sweden) and SKOV-3—human-derived ovarian cancer (ATCC HTB-77, Wesel, Germany). Two different toxicity assays were used which were Cell Counting Kit-8 (CCK-8, Cat.# 96922, Sigma Aldrich, Stockholm, Sweden) and NucGreen^®^ viability assay (NucGreen™ Dead 488 ReadyProbes™, Cat# R37109, Thermo Fisher, Stockholm, Sweden). All the tests were performed following the supplier’s instructions; the absorbance (CCK8) and fluorescence (NucGreen^®^) measurements were performed on a Hidex Sense multi plate reader (Kem-En-Tec Nordic, Uppsala, Sweden). The viability of the cells exposed to NPs is normalized to the viability of the non-exposed cells (the negative control). 

Dulbecco’s modified Eagle medium (DMEM, Sigma Aldrich) containing 10% fetal bovine serum (FBS) was used as cell culture medium. As negative control, cells grown in the absence of NPs were used. The cells were about 80% confluent when the assays were performed. 

The cells were split and seeded into 96-well plates (Cat. # 167008, Thermo Fisher, Stockholm, Sweden), corresponding to 30,000 to 35,000 cells per well in a 96-well plate, one day before NP exposure. The cells were exposed for 24 h to a dilution series of Rh NPs with different morphologies. The concentration of NPs tested was highest at 250 μg/mL in the first well, and further dilution series were obtained by a 2-fold dilution (as 125, 62.5, and 31.25 μg/mL) of the stock to obtain a concentration series. Only in the case of the Rh_PVP-CTAC sample, the highest concentration was 100 μg/mL in the first well, and further dilution series were obtained by a 2-fold dilution (as 50, 25, and 12.5 μg/mL) of the stock. 

The CCK-8 assay, in brief, was performed by adding the substrate, 10 µL WST-8 (2-(2-methoxy-4-nitrophenyl)-3-(4-nitrophenyl)-5-(2,4-disulfophenyl)-2H-tetrazolium, monosodium salt), to 100 µL cell media/well. The plates were incubated at 37 °C, 5% CO_2_ for 2 h, after which the absorbance was measured. During incubation, the substrate was reduced by the enzymes in active cells to an orange formazan dye directly proportional to the number of living cells. To avoid interference, the media were aspirated and new fresh media were added just before the initiation of the assay. 

NucGreen^®^ is a cell-impermeant stain that emits bright green (535 nm) fluorescence when bound to DNA. Cells that have lost plasma membrane integrity are stained within minutes, making NucGreen^®^ a stain used to estimate live/dead cell ratios. For the NucGreen viability assay, 2 drops of NucGreen reagent were added per mL of medium and after 5 min incubation, the fluorescence signal was detected at 535 nm, with an excitation wavelength of 485 nm [20].

### 2.5. XFCT Phantom Experiments 

The performance of the synthesized NPs as contrast agents in small-animal XFCT was investigated in an in situ imaging setting. A pipette tip (Eppendorf epTIPS, 2–200 μL) was used as an imaging target by filling it with a Rh-PVP NPs sample dispersion in DI water at a concentration of 250 μg/mL Rh. The pipette tip was then surgically inserted in a sacrificed mouse for another study (15-week-old, female, NOD.Cg-Prkdcscid Il2rgtm1Sug/JicTac, Taconic Biosciences, Lille Skensved, Denmark). The mouse was positioned inside a 60 mL tube and imaged using our in-house preclinical XFCT arrangement [21]. The choice of the pipette tip as an imaging target allowed, due to its conical shape with a decreasing cross-sectional diameter, an investigation of the smallest observable feature size at the fixed NP concentration. The inner diameter of the pipette tip ranged from 0.5 to 4 mm along its length of 35 mm.

Simultaneous XFCT and computed tomography (CT) was performed on the sacrificed mouse by acquiring 30 projection images over 180 degrees. Each projection image was acquired with steps of 200 μm and exposure time of 10 ms, resulting in a total scan time of ~1.5 min for each axial slice (of 200 μm thickness). We note that the chosen acquisition settings and the radiation dose, which is estimated to be ~25 mGy, have been demonstrated suitable for in vivo imaging in our recent study [21]. The acquired CT data were reconstructed using a standard filtered back-projection algorithm, while the acquired XFCT data were reconstructed using an in-house developed iterative algorithm. For more details on the imaging arrangement, see Ref. [21].

## 3. Results and Discussion

Formation of metallic NPs in polyol synthesis is rather straightforward, where metal ions are reduced by the polyalcohol solvent, in our case EG. PVP acts as a pooling agent, by forming reservoirs of metal ions. In this respect, its concentration can play an important role on the size of nucleated NPs. Parameters such as reaction temperature during nucleation and crystal growth, reaction time, solvent type, and concentration of precursors and capping agents can affect the shape of final particles. In this study, we investigated the effect of different additives (PVP, KBr, CTAB, CTAC, Na-Ac, and Na-Cit) on the NP morphology while keeping the other parameters unaltered. Details of the prepared samples are presented in Table 1.

### 3.1. Morphology Analysis 

When all the others synthesis parameters of the reaction are kept the same, the morphological variations of NPs are dependent on the type of additives used with the anionic ligands inducing the strongest morphology variation in the synthesis of NPs. For metals with a face-centered cubic (fcc) crystal structure (including Rh), the surface energy (γ) follows the trend γ(111) < γ(100) < γ(110) with the highest surface energy in the (110) facet. These relative energies can be modified by surface-adsorbing species exposing facets that would not normally be thermodynamically favored, introducing a degree of anisotropy into the NP geometry [22]. 

**Figure 1:**
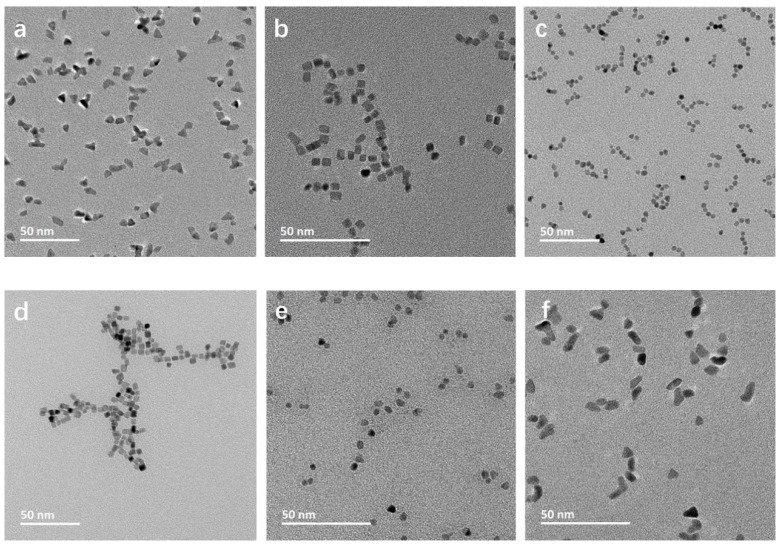
TEM micrographs of (**a**) Rh_PVP; (**b**) Rh_PVP-KBr; (**c**) Rh_PVP-Na-Ac; (**d**) Rh_ PVP-CTAB; (**e**) Rh_PVP-Cit; and (**f**) Rh_PVP-CTAC.

As expected, the morphology of the obtained Rh NPs varies greatly dependent on the additives present during the synthesis (Table 1, Figure 1). With only PVP added, predominantly triangular NPs are observed (Figure 1a), revealing anisotropy due to the adsorption of PVP on the NP surface. Br-containing additives, KBr (Figure 1b) and CTAB (Figure 1d), resulted in cubic NPs. Among the carboxyl group-containing additives, acetate (NaAc, Figure 1c) and citrate (NaCit, Figure 1e) ions led to the formation of spherical NPs, which is indicative of isotropic growth, eliminating the effect of PVP. Rh NPs synthesized by the addition of CTAC (Figure 1f) display an irregular mixed polygonic particle morphology. Cl^−^ ions did not show the same effect as the one observed in the presence of Br^−^ ions. The resultant NPs present a mixed polygon morphology, including trigonal, similar to the ones synthesized in the presence of PVP only. The mixed polygon morphology may be due to a complex formation between the additional Cl^−^ ions and Rh, influencing the NP growth dynamics [23].

**Figure 2:**
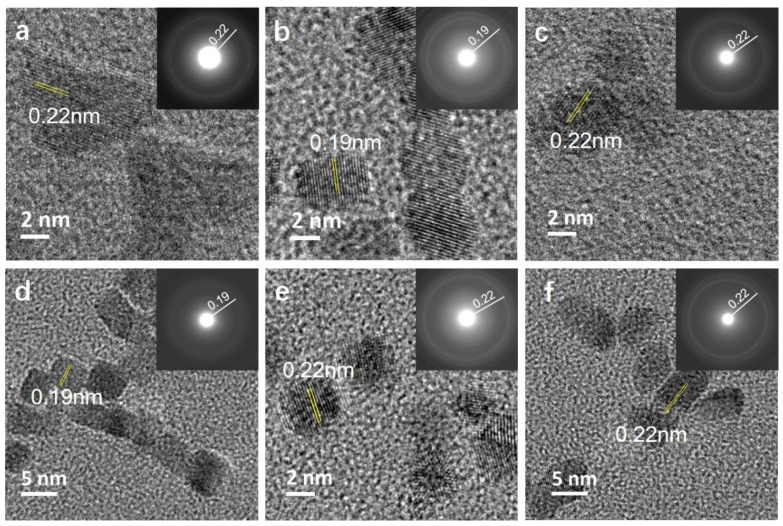
(**a**–**f**) HRTEM micrographs of Rh NPs: (**a**) Rh_PVP; (**b**) Rh_PVP-KBr; (**c**) Rh_PVP-Na-Ac; (**d**) Rh_ PVP-CTAB; (**e**) Rh_PVP-Cit; and (**f**) Rh_PVP-CTAC (the lattice spacings are indexed for fcc Rh using ICDD PDF card no: 03-065-2866).

The observed morphological differences are related to the preferential adsorption of anions on certain facets with the NP growth allowed predominantly along them. One common feature observed in the HRTEM micrographs of the samples (Figure 2) is the fact that the NPs obtained in the presence of all studied anions are single crystalline, with the measured d values matching the fcc Rh (ICDD PDF card no: 03-065-2866). Lattice fringes of Rh samples synthesized in the presence of PVP (Figure 2a), Na-Ac (Figure 2c), Na-Cit (Figure 2e), and CTAC (Figure 2f) have a d value of 0.22 nm, which matches well with the (111) plane of fcc-Rh. The additives containing Br^−^ ions, however, showed a slightly different passivating behavior. Br- ions are reported to passivate the (100) plane in Rh nanocrystals [14]. Our observations confirmed the stabilization of the (100) surface when Br^−^-bearing KBr (Figure 2b) and CTAB (Figure 2d) are used as additives. In both cases, the NPs have a cubic morphology with lattice fringes of 0.19 nm corresponding to the (200) plane of Rh, proving that Br^−^ ions are capable of getting adsorbed on the surface of the NPs formed, even in the presence of PVP.

Plasmonic NPs of Ag and Au show a size- and morphology-dependent UV–Vis absorption behavior [24]. In order to study the effect of morphology on Rh NP absorption characteristics, we performed UV–Vis analysis on the synthesized samples. UV–Vis absorption spectra indicated that the maximum absorption wavelength due to the surface plasmon resonance (SPR) of Rh varied with particles morphology, with a peak value in the range of 210–220 nm (Figure S1). The intensity of SPR absorption is the highest for Rh NPs with a cubic morphology (Rh_PVP-KBr and Rh_PVP-CTAB), with an absorption edge at 225 nm. A similar trend of absorption intensity and edge was observed for triangular NPs (Rh_PVP). Spherical NPs (Rh_PVP-Ac and Rh_PVP-Cit) display an absorption maximum at 210 nm, with an absorption edge at about 220 nm. All these samples showed a weak, broad absorption at the 270–275 nm region. Polygonic Rh NPs (Rh_PVP-CTAC) showed fluctuating absorption in the range of 200–225 nm, followed by a broad absorption centered at 275 nm, with a very high background absorption. This may be due to the polydispersity in the shapes of NPs (observed in Figure 1f) increasing the background absorption significantly.

### 3.2. Particle Size and Surface Chemistry Analyses 

**Figure 3:**
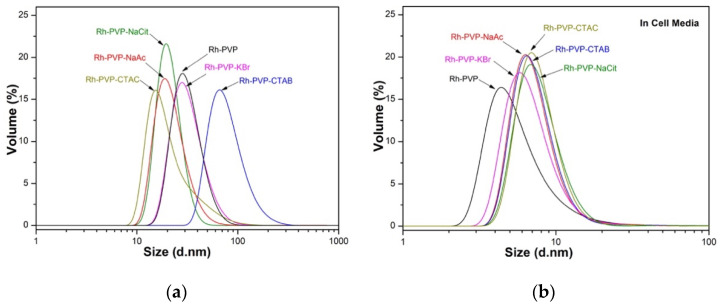
Hydrodynamic size distribution plots, using the DLS technique, of as-synthesized Rh NPs, with different morphologies in (**a**) DIW and (**b**) DMEM. Data presented in volume distribution.

Besides the morphological effects on the Rh NPs, the dry particle size obtained from TEM micrographs, hydrodynamic size, and surface properties are also influenced by the additives used (Table 2, Figure 3). The largest NPs were formed when PVP only (7.6 nm) and PVP-CTAC (6.6 nm) are used. Bromide-containing additives, KBr and CTAB, formed NPs with an average size of 4.7–4.8 nm. Carboxylic acid functionality-bearing additives, Na-Ac and Na-Cit, resulted in NPs with an average size of 3.1–3.4 nm. The differences in NP size are ascribed to the fact that the additives clearly affect the crystal nucleation and growth kinetics in addition to directing the morphology. It may be reasonable to relate this to the notion that acetate and citrate ions allowed a fast nucleation, i.e., burst nucleation, and bromide ions constrained the growth moderately, whereas PVP only and chloride ions yielded the largest size of Rh NPs due to the impeded kinetics allowing particle ripening. 

The NPs’ dispersed sizes, i.e., the hydrodynamic size, were evaluated in DIW and cell culture media-DMEM, and the results are presented graphically in Figure 3 and summarized in Table 2. Rh NPs synthesized with PVP, PVP-Cit, and PVP-Ac have an average hydrodynamic size of around 30 nm, the NPs synthesized in the presence of PVP-KBr and PVP-CTAC have an intermediate size of 40–50 nm and the size of about 100 nm was found when PVP-CTAB was used. The polydispersity index (PDI), a parameter that defines the goodness of size distribution, where a value less than 0.3 relates to a homogeneous population [25], is lower than 0.3 for all samples except the PVP-CTAC and PVP-Ac samples. As a general trend, all the Rh NPs synthesized in this work present a very low surface charge (Table 2), under (±) 30 mV. Despite the low charge density on their surface, the NPs are stable in solution probably due to the steric hindrance exerted by the polymeric chains attached to the surface. Rh_PVP NPs show near-zero surface charge at neutral pH. A common trend for all other samples is the net negative surface charge on the NPs. This correlates well with the morphological differences being controlled by the adsorbed anions on the NPs. It is important to note that the size distributions of all synthesized Rh NPs, regardless of the shape, are suitable for use in XFCT bio-imaging.

Particle size distribution is also measured upon dispersing the NPs in DMEM, containing 10% FBS. Results indicate a smaller dispersed size, except Rh_PVP-Cit, with an increase in the polydispersity index to the 0.4–0.6 level. The ionic strengths of DMEM could have helped the decrease initially observed in NP clusters, while various proteins in DMEM led to larger agglomerate formations, which are detected when scattering intensity is used for hydrodynamic NP size distribution (see ESI, Figure S2). Adsorption of proteins is also influential in the final surface charge of Rh NPs in DMEM, which are observed to have enhanced their negative charge as compared to their surface charge in DIW (Table 2).

**Figure 4:**
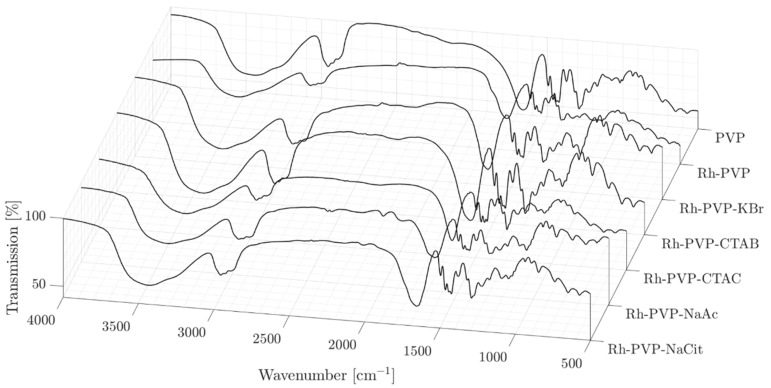
FT-IR spectra of pure PVP and Rh NPs; Rh_PVP, Rh_PVP-KBr, Rh_PVP-CTAB, Rh_PVP-CTAC, Rh_PVP-Ac, and Rh_PVP-Cit.

The FT-IR spectra reveal that the surface of all Rh NPs shows dominantly PVP features with no strong indication of the presence of secondary additives (Figure 4), probably due to the easy removal of these additives during the washing steps. A common feature is the shift of the position of the C = O absorption band from 1662 for the pure PVP to around 1650 cm^−1^ for all NPs synthesized, indicating the conjugation of PVP via the carbonyl group onto the Rh NPs’ surface. The quantitative determination of the organic material proportion in the samples by thermal gravimetric analysis (TGA) showed that the degradation of the organics was completed at 500 °C (ESI, Figure S3). The thermograms and a table summarizing the findings are presented in Figure S3. The Rh_PVP system showed the highest NP content of about 13 wt%, where the NP content decreased to 6 wt%, in a non-monotonic way for the other samples.

### 3.3. Cytotoxicity Studies

The cellular viability as an indicator for the toxicity potency of an agent can be affected in various ways. We, therefore, tested the membrane integrity (fluorescence NucGreen assay) and cellular metabolic activity (colorimetric CCK-8 assay) of cells exposed to Rh NPs with different morphologies in a concentration-dependent manner, with the highest dose of 250 μg/mL and the lowest nominal dose of 30 μg/mL for the 24-h exposure time. It is known that different cell types will react in a distinct way in the presence of external stimuli. Therefore, in this work the cytotoxicity of Rh NPs was evaluated on murine macrophages (RAW264.7) and a human ovarian cancer (SKOV-3) cell line. The RAW264.7 macrophages serve as a model for immune cells, while SKOV-3 is used as a model of tumoral cells to investigate if the exposure to NPs will induce a different level of toxicity in different cell lines. The half maximal inhibitory concentration (IC50) is specified for NPs, whenever relevant, in various toxicity assays against different cell lines. 

NucGreen is staining only the cells that have lost plasma membrane integrity. The membrane integrity of RAW 264.7 is not compromised when exposed to trigonal Rh NPs (Rh_PVP). However, it is significantly compromised when exposed to cubic NPs (Rh_PVP-KBr and Rh_PVP-CTAB), at all doses. In the case of spherical NPs (Rh_PVP-NaAc (IC50: 118 μg/mL) and Rh_PVP-NaCit (IC50: 120 μg/mL)), the membrane integrity is affected in a concentration-dependent manner, the viability increasing with the decreasing concentration of NPs. Cell viability is significantly reduced for doses ≥ 125 μg/mL (ESI, Figure S4). Rh NPs with a mixed polygon morphology (Rh_PVP-CTAC), presented separately in Figure S6, due to their lower concentration reaching a maximum of 100 μg/mL, showed high viability at all the doses tested. 

**Figure 5:**
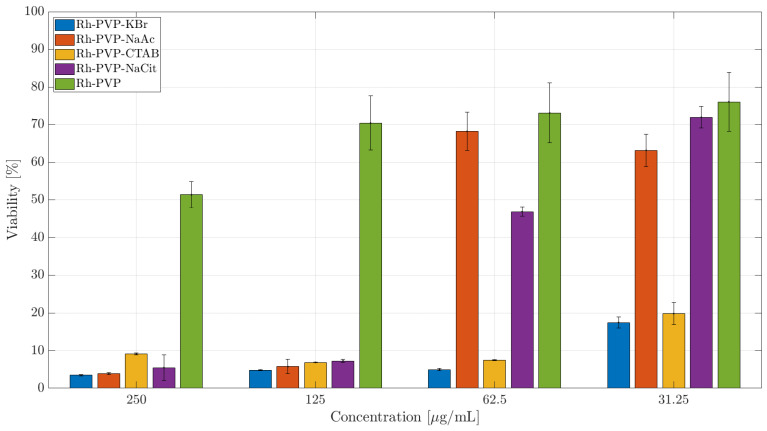
CCK-8 toxicity assay of Rh-NPs in the RAW 264.7 cell line after 24 h of incubation. The percentage of cell viability is calculated relative to the cells incubated in the absence of NPs (negative control) with 100% viability.

**Figure 6:**
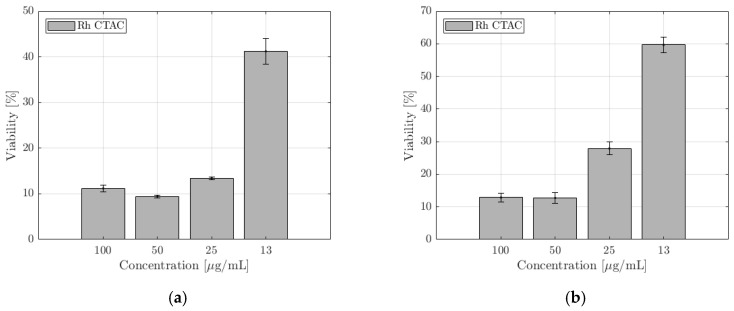
CCK-8 toxicity assays of Rh-CTAC NPs in the (**a**) RAW 264.7 and (**b**) SKOV-3 cell lines after 24 h of incubation. The percentage of cell viability is calculated taking negative control cells incubated in the absence of NPs with 100% viability.

The exposure to triangular Rh NPs (Rh_PVP) of the RAW264.7 cell line in doses up till 125 μg/mL did not significantly decrease the cells’ metabolic activity, with the viability reducing to about 50% at a dose of 250 μg/mL (Figure 5. Among the spherical NPs, Rh-Cit NPs (IC50: 56 μg/mL) reached viability beyond 60% when the dose was reduced to 60 μg/mL, while Rh-Ac NPs (IC50: 86 μg/mL) showed viability of >70% at the lowest dose of 30 μg/mL. Cubic NPs (Rh_PVP-KBr and Rh_PVP-CTAB) showed very low viability, or a high level of toxicity, at all the doses tested. NPs with a mixed polygon morphology (Rh-CTAC, Figure 6) showed a high level of toxicity in the RAW264.7 cell line.

The cytotoxicity of some organic surface-coating agents used for NP synthesis has been studied in the literature, where the cationic surfactant CTAB was reported to be highly toxic [26]. In the case of Rh NPs synthesized in the presence of CTAB and CTAC, the residue of the cationic part could cause the observed negative effect. The common character of Rh NPs synthesized in the presence of KBr and CTAB is their cubic morphology, due to surface-adsorbed Br- ions, which resulted in high cytotoxicity levels. Br^−^ ions are known to exist in humans and animals and can reach up to almost half of the Cl^−^ content in red blood cells. [27] Adsorbed inorganic Br^−^ is, therefore, not expected to show the observed cytotoxicity in the case of KBr-assisted synthesis. Therefore, it is reasonable to ascribe the observed cytotoxicity to the cubic NP morphology. In general, SKOV-3 cells are more resistant to external agents. Indeed, the membrane integrity of SKOV-3, maintained at >80%, is not compromised significantly (Figure S5), when exposed to trigonal (Rh_PVP) and spherical NPs (Rh_PVP-NaAc and Rh_PVP-NaCit). However, in the presence of NPs with a cubic morphology (Rh_PVP-KBr (IC50: 119 μg/mL) and Rh_PVP-CTAB (IC50: 115 μg/mL)) in doses ≥ 125 μg/mL, the viability is significantly reduced. NPs with a mixed polygon morphology (Rh_PVP-CTAC) (Figure S6) showed high viability at all the doses tested, similar to the response of macrophages. 

**Figure 7:**
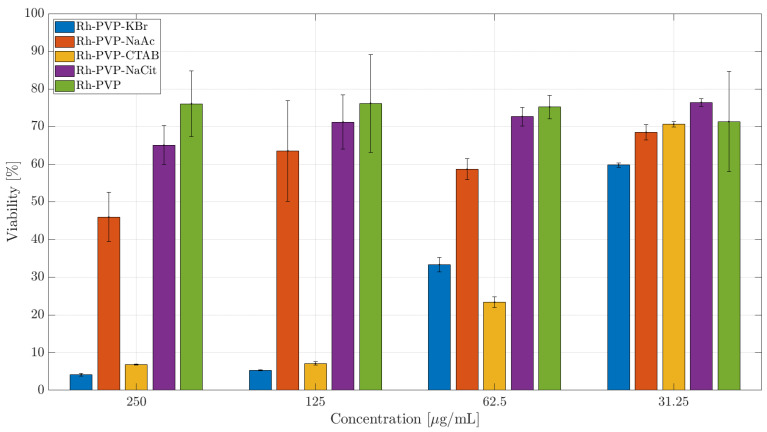
CCK-8 toxicity assay of Rh NPs in the SKOV-3 cell line after 24 h of incubation. The percentage of cell viability is calculated relative to the cells incubated in the absence of NPs (negative control) with 100% viability.

A similar trend is observed in the CCK-8 assay (Figure 7) with the trigonal NPs (Rh-PVP) exerting the lowest toxicity followed by spherical NPs (RhPVP-NaCit and Rh-PVP-NaAc (IC50: 218 μg/mL)). The highest toxicity level is observed when the cells were incubated with cubic NPs (Rh_PVP-CTAB (IC50: 42 μg/mL) and Rh_PVP-KBr (IC50: 41 μg/mL)). NPs with a mixed polygon morphology (Rh_PVP-CTAC) (Figure 6b) showed a high level of toxicity in the SKOV-3 cell line at higher doses, reaching a viability of >60% at the lowest dose of 13 μg /mL NP concentration.

The differentiated cytotoxicity response of the macrophage cell line (RAW 264.7) and cancer cell line (SKOV-3) in the presence of the NPs might be explained by the different functions the macrophages and cancer cells are specialized to perform. While the macrophages should react to any external or internal aggression factors, the cancer cells are programed to adapt to any conditions in order to survive and multiply. This work showed that membrane integrity and metabolic activity, expressed as viability, are influenced to a similar extent, for both the cell lines, with respect to the morphology of Rh NPs. Particles with a mixed polygon morphology showed the highest negative impact on cellular activity, followed by cubic and spherical NPs. Trigonal NPs showed the lowest negative effect, or the highest viability, among the NP series. From the FT-IR analysis, we see no significant evidence of the presence of functional groups, especially for surfactants CTAB and CTAC. Trace amounts of these functional groups may still reside on the surface dominating PVP, or at the immediate NP surface. By looking at the significantly different response of cellular activity towards these NPs, it is reasonable to ascribe the observed effects to the NP morphology. 

Similar observations were reported for noble metal NPs. For instance, gold nanorods prepared using CTAB were shown to be highly toxic to human skin cells due to the presence CTAB, not the gold nanorods themselves [28]. It was later shown that over-coating gold nanorods with polymers substantially reduced their cytotoxicity [29]. If any of the Rh NP morphologies showing a high level of toxicity prove to be more interesting candidates for biomedical applications, their surface chemistry can be further modified using polymers or other inorganic coatings, such as silica, to improve their biocompatibility, without adversely influencing the diagnostics function.

### 3.4. XFCT Performance

**Figure 8:**
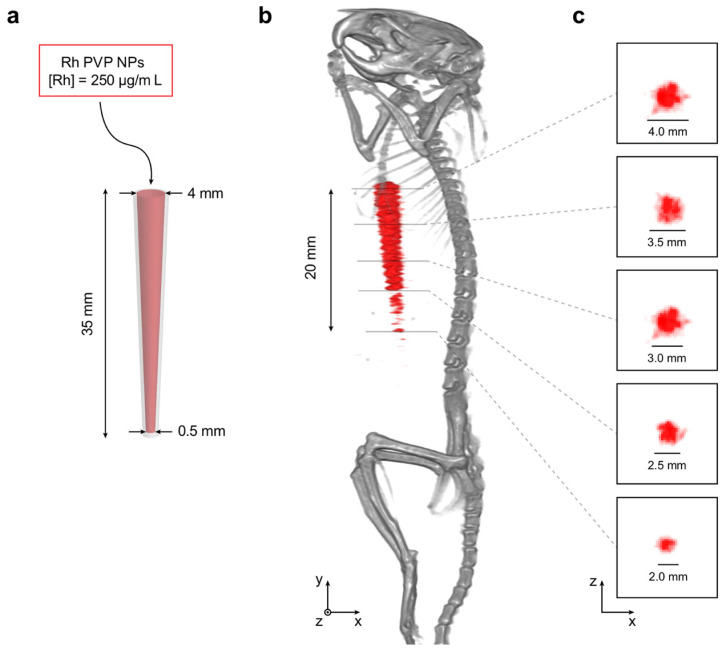
In situ XFCT performance of sample Rh-PVP. (**a**) A pipette tip was used as an imaging target by filling it with Rh-PVP NPs (250 μg/mL), where the conical shape offered a target with an inner diameter ranging from 4 to 0.5 mm. (**b**) Reconstructed tomographic data visualized in 3D (grayscale: CT, red: XFCT). The portion of the pipette tip visible in the XFCT reconstruction was estimated to be ~20 mm. (**c**) Selected axial slices with 5 mm separation in y of the XFCT reconstruction, with the theoretical expected diameters at each location denoted with scale bars and numbers. Each pixel in the axial slices corresponds to 200 × 200 μm^2^.

Figure 8 shows the in situ small-animal XFCT imaging experiment. Using the pipette tip (Figure 8a) as an imaging target allowed the investigation of the smallest observable feature size at the fixed NP concentration, due to its conical shape with a decreasing cross-sectional diameter. The content of the pipette tip could not be separated and visualized in the CT reconstruction, while it could be clearly visualized in the XFCT reconstruction (c.f., Figure 8b). For diameters of the pipette tip between 4 and 2.5 mm, the NP content could be reliably reconstructed, while for diameters smaller than 2.5 mm, it became increasingly difficult for the reconstruction algorithms to separate the XRF signal from background X-ray scattering. Below the 2 mm diameter mark, the reconstruction algorithms could no longer separate the XRF signal from the background. These results agree with previous simulations, which indicated that a local NP concentration of >1 mg/mL is necessary to reach sub-mm feature visualization [30]. 

Selected cross-sections of the XFCT reconstruction are shown in Figure 8c and compared to the theoretical inner diameters of the pipette tip at the selected locations. We note that estimated cross-sectional pipette tip diameters in the XFCT reconstruction are in the range of theoretical values between 4 and 2 mm, and conclude, therefore, that few-mm features with a local NP concentration in the 100 μ g/mL range can be visualized using XFCT. In other words, this type of in situ imaging experiment offers a controlled environment to investigate relations between parameters such as feature size and observable local Rh NP concentration, which is not directly possible in in vivo scenarios.

## 4. Conclusions

Rh nanoparticles (NPs) with various morphologies were synthesized in the presence of a large palette of additives during the polyol synthesis process. The halide salts and surfactant additives including sodium acetate, sodium citrate, KBr, CTAB, and CTAC, in addition to PVP, showed a tuning effect on the shape of the Rh NPs’ morphology. When PVP was used as the only additive, trigonal NPs were obtained. Additives with Br^−^ ions (CTAB and KBr) resulted in NPs with a cubic morphology, while those with carboxyl groups (sodium citrate and acetate) formed spheroid NPs. Cl^−^ ions (CTAC) resulted in a mixture of polygon morphologies. The observed cytotoxicity response evaluated using two different viability assays, on macrophages and ovarian cancer cell lines, shows a strong dependence on the NPs morphology. The cells exposed to trigonal Rh NPs showed the highest viability, among the NP series. Particles with a mixed polygon morphology showed the highest cytotoxicity, followed by cubic and spherical NPs, which can be correlated with a more shape-dependent response triggering. The performance of Rh NPs as contrast agents for small-animal XFCT was demonstrated in an in situ experiment, showing promise for future in vivo imaging. This work provides a detailed route for the synthesis, morphology control, and characterization of Rh NPs as viable contrast agents for XFCT bio-imaging.

## Figures and Tables

**Table 1:** Summary of Rh nanoparticles (NPs) with diverse morphologies, synthesized using various additives.

Sample Designation	Precursor	Solvent	Additive	Stabilizer	Morphology
Rh_PVP	RhCl_3_	EG	-	PVP	triangle
Rh_PVP-KBr	RhCl_3_	EG	KBr	PVP	cubic
Rh_PVP-CTAB	RhCl_3_	EG	CTAB	PVP	cubic
Rh_PVP-CTAC	RhCl_3_	EG	CTAC	PVP	polygon
Rh_PVP-NaAc	RhCl_3_	EG	NaAc	PVP	spherical
Rh_PVP-NaCit	RhCl_3_	EG	NaCit	PVP	spherical

**Table 2:** Dry size (TEM size ± standard deviation (SD)), hydrodynamic size (DLS size, the polydispersity index (PDI)), and zeta potential of Rh NPs with different morphologies in DIW and DMEM with 10% FBS.

		In DIW		In CCM + 10%FBS	
Sample	TEM Size; Mean ± SD (nm)	DLS Size [PDI] (nm)	Zeta Potential (mV)	DLS Size [PDI] (nm)	Zeta Potential (mV)
**Rh-PVP**	7.6 ± 1.6	29.1 [0.23]	0.18	18.1 [0.49]	−9.72
**Rh-PVP-KBr**	4.7 ± 0.8	41.1 [0.23]	−7.74	16.5 [0.46]	−11.24
**Rh-PVP-CTAB**	4.8 ± 0.7	97.1 [0.19]	−11.57	37.8 [0.64]	−8.76
**Rh-PVP-CTAC**	6.6 ± 1.3	46.2 [0.39]	−3.59	19.7 [0.55]	−5.69
**Rh-PVP-NaAc**	3.4 ± 0.7	31.9 [0.41]	−9.43	17.8 [0.44]	−10.02
**Rh-PVP-NaCit**	3.1 ± 0.6	28.3 [0.18]	−5.05	50.7 [0.68]	−9.38

## Supplementary Materials

The following are available online at https://www.mdpi.com/2079-4991/10/11/2129/s1, Figure S1: UV–Vis absorption spectra of Rh NPs; Rh_PVP, Rh_PVP-KBr, Rh_PVP-Ac, Rh_PVP-CTAB, Rh_PVP-Cit, and Rh_PVP- CTAC. Figure S2: Hydrodynamic particle size distribution plots, using DLS, of as-synthesized Rh NPs, with different morphologies. Data presented in intensity distribution to show the agglomerates formed. Figure S3: TGA thermograms of Rh NPs; Rh_PVP, Rh_PVP-KBr, Rh_PVP-CTAB, Rh_PVP-CTAC, Rh_PVP-Ac, and Rh_PVP-Cit. Figure S4. NucGreen toxicity assay of Rh NPs in the RAW 264.7 cell line after 24 h of incubation. The percentage of cell viability is calculated relative to the cells incubated in the absence of NPs (negative control) with 100% viability. Figure S5. NucGreen toxicity assay of Rh NPs in the SKOV-3 cell line after 24 h of incubation. The percentage of cell viability is calculated relative to the cells incubated in the absence of NPs (negative control) with 100% viability. Figure S6. NucGreen toxicity assays of Rh-CTAC NPs in (a) RAW 264.7 and (b) SKOV-3 cell lines after 24 h of incubation. The percentage of cell viability is calculated taking negative control cells incubated in the absence of NPs with 100% viability.
