# Supplementary material for: Synthesis, Physicochemical Characterization, and Cytotoxicity Assessment of Rh Nanoparticles with Different Morphologies-as Potential XFCT Nanoprobes
Source: Nanomaterials (Basel). 2020 Oct 27;10(11):2129. doi: 10.3390/nano10112129 (PMC7692549; doi:10.3390/nano10112129)
Supplement: Supplementary file 1 [file nanomaterials-10-02129-s001.pdf]

Electronic Supplementary Material
for

Synthesis, Physicochemical Characterization, and Cytotoxicity Assessment of Rh Nanoparticles with Different Morphologies -as Potential XFCT Nanoprobes

Yuyang Li, Giovanni M. Saladino, Kian Shaker, Martin Svenda, Carmen Vogt, Bertha Brodin, Hans M. Hertz, Muhammet S. Toprak*

Biomedical and X-Ray Physics, Department of Applied Physics, KTH Royal Institute of Technology, Stockholm, Sweden

* Correspondence: toprak@kth.se (MST); Tel.: +46-735-519358

UV-Vis Analysis

UV-vis absorption spectra indicated that the maximum absorption wavelength due to the surface plasmon resonance (SPR) of Rh varied slightly with particles morphology, with a peak value in the range of 210–220 nm (figure S1) and an absorption band edge at 225 nm.

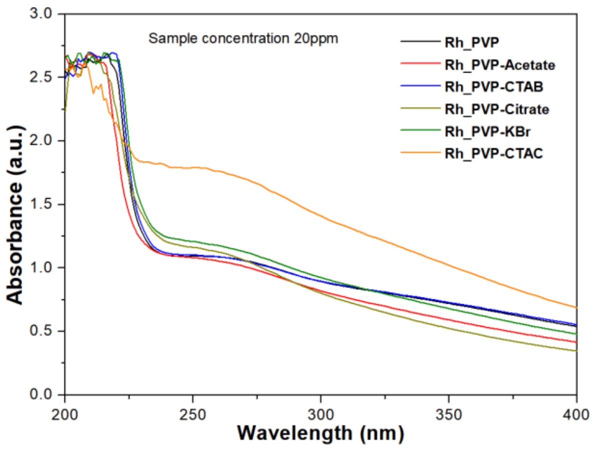

Figure S1. UV-vis absorption spectra of Rh NPs; Rh_PVP, Rh_PVP-KBr, Rh_PVP-Ac, Rh_PVP-CTAB, Rh_PVP-Cit and Rh_PVP- CTAC. Rh concentration is kept constant (20 ppm) in the measured samples.

Particle Size Analysis

Particle size distribution is studied using the DLS system, where the signal is obtained from the scattering of light with particles with different hydrodynamic radius. Typically, we prefer to represent the data in the *volume distribution* as it is a more appropriate representation for the NPs to be used for biomedical applications. The light intensity is inversely proportional to the particle size, making it hard to estimate the proper hydrodynamic size from polydisperse NP sample populations. Hydrodynamic size distribution of Rh NPs with various morphology in CCM is therefore presented here in *intensity distribution* (figure S2), in order to clearly show the heterogeneity of the distribution. The observed agglomeration is most likely caused by the adsorption of proteins on the surface of Rh NPs.

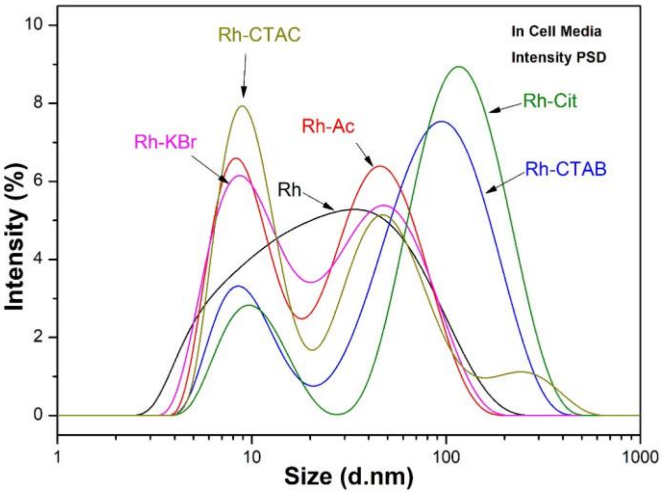

Figure S2. Hydrodynamic particle size distribution plots, using DLS, of as-synthesized Rh NPs, with different morphologies. Data presented in intensity distribution to show the agglomerates formed.

Thermal Gravimetric Analysis

The quantitative determination of the organic material proportion in the samples by TGA showed that the degradation of the organics was completed at 600 ^{o}C (figure S3). Therefore, the residual weight, thus the ratio of organic to inorganic content can be reasonably estimated from this temperature point. Rh NPs synthesized using only PVP stabilizer yielded 13 wt% inorganic content, followed by 10 wt% for PVP-CTAB system, 9 wt% for Rh-PVP-NaCit, 7 wt% for Rh-PVP-NaAc and finally 6 wt% for Rh-PVP-KBr. The variation in content of PVP is correlating to the size of the NPs. As the size of NPs decreases their surface area per unit weight increases, which will reflect in a larger quantity of stabilizers adsorbed as compared to the bigger particles. The trends

observed for the NPs synthesized in the presence of different additives correlate partially with the particle size, where the highest inorganic content -or the lowest organic content, is observed for the largest particles in the series (Rh-PVP) followed by the next larger NP (Rh_PVP-CTAB). For NP sizes lower than 6 nm no clear trend is observed, perhaps due to their agglomeration and interparticle entrapment of residual chemicals.

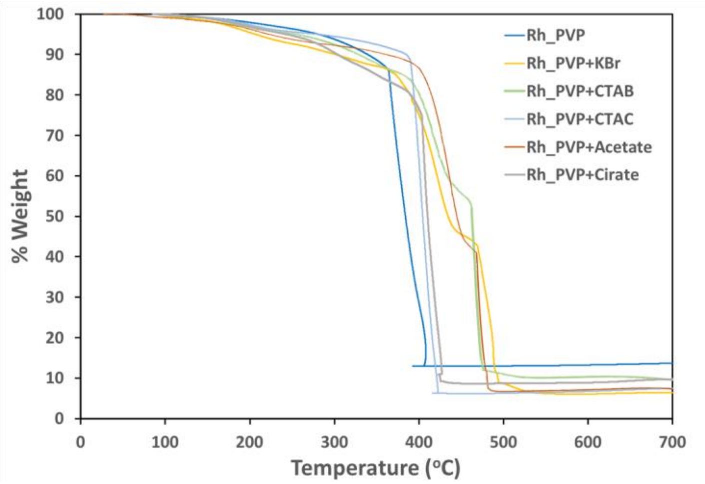

Sample	Rh NP wt%
Rh-PVP	13
Rh-PVP-KBr	6
Rh-PVP-CTAB	10
Rh-PVP-CTAC	8
Rh-PVP-NaAc	7
Rh-PVP-NaCit	9

Figure S3. TGA thermograms of Rh NPs; Rh_PVP, Rh_PVP-KBr, Rh_PVP- CTAB, Rh_PVP-CTAC, Rh_PVP-Ac, and Rh_PVP-Cit.

Toxicity Assay Using NucGreen

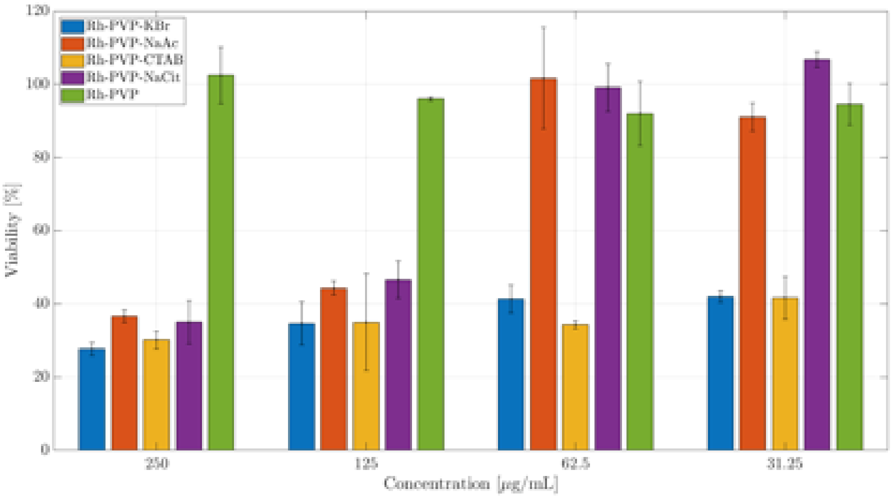

Figure S4. NucGreen toxicity assay of Rh NPs in RAW 264.7 cell line after 24 h incubation. The percentage of cell viability is calculated relative to the cells incubated in the absence of NPs (negative control) with 100% viability.

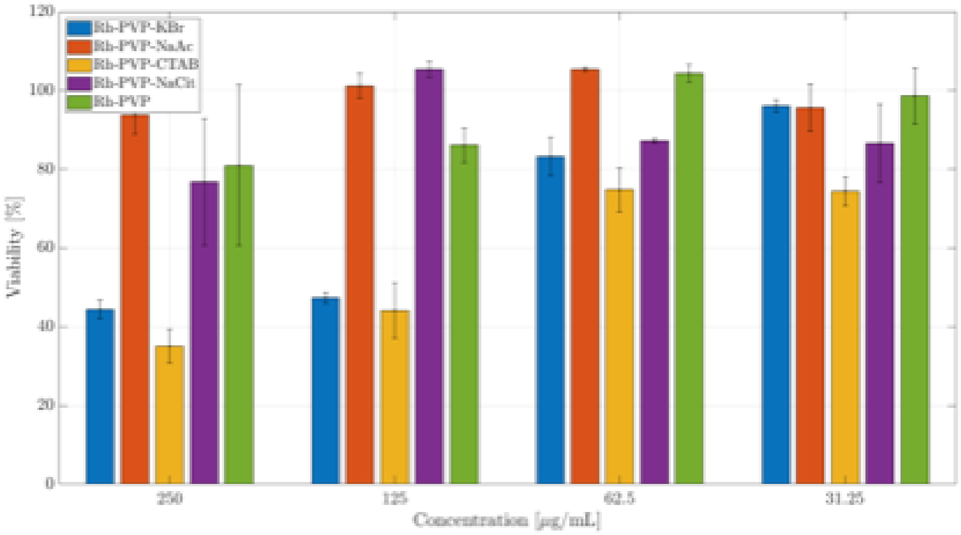

Figure S5. NucGreen toxicity assay of Rh NPs in SKOV-3 cell line after 24 h incubation. The percentage of cell viability is calculated relative to the cells incubated in the absence of NPs (negative control) with 100% viability.

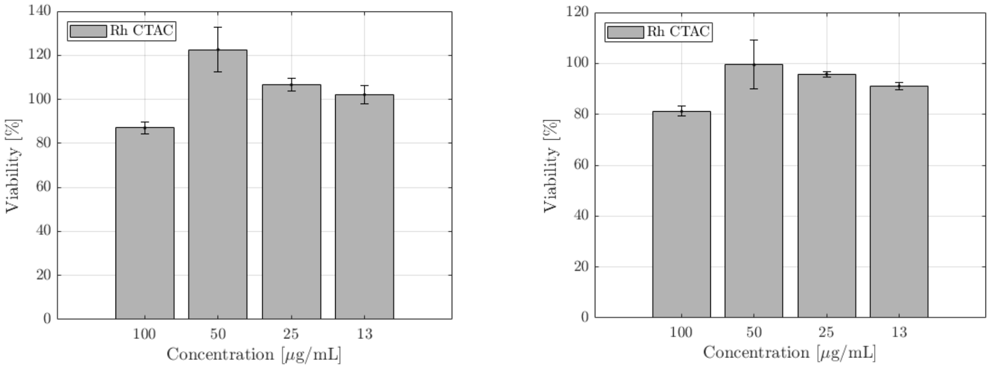

(a) (b)

Figure S6. NucGreen toxicity assays of Rh-CTAC NPs in (**a**) RAW 264.7 and (**b**) SKOV-3 cell lines after 24 h incubation. The percentage of cell viability is calculated taking negative control cells incubated in the absence of NPs with 100% viability.
